# Standardization of an experimental model suitable for studies on the effect of exercise on arthritis

**DOI:** 10.1590/S1679-45082013000100014

**Published:** 2013

**Authors:** Raquel Pinheiro Gomes, Elisangela Bressan, Tatiane Morgana da Silva, Monique da Silva Gevaerd, Carlos Rogério Tonussi, Susana Cristina Domenech

**Affiliations:** 1 Universidade do Estado de Santa Catarina, Florianópolis, SC, Brazil; 2 Universidade Federal de Santa Catarina, Florianópolis, SC, Brazil

**Keywords:** Arthritis, experimental, Edema, Models, animal, Nociception, Freund's adjuvant/administration & dosage, Exercise, Wistar

## Abstract

**Objective:**

To standardize an experimental model of chronic monoarthritis induced by complete Freund's adjuvant appropriate for the analysis of the effect of walking on nociception and on joint edema.

**Methods:**

The following factors were evaluated as to monoarthritis induction: route and site of administration, number and interval of inoculations, *Mycobacterium* species, and animal gender. Wistar male and female rats (200 to 250g) received two injections of complete Freund's adjuvant containing *Mycobacterium tuberculosis* (1.0mg/mL; 50*μ*L) or *Mycobacterium butyricum* (0.5mg/mL; 50*μ*L) intra-articularly in the tibiotarsal or tibiofemoral joints, or an injection of complete Freund's adjuvant (*Mycobacterium butyricum* or *tuberculosis*) intradermally at the base of the tail and another intra-articularly (tibiotarsal or tibiofemoral). The animals were submitted to evaluations of articular disability and edema. Articular disability was assessed by paw elevation time (in seconds) during the one-minute walk test. Edema of the tibiofemoral joint was assessed by variation of joint diameter (cm). Tibiotarsal joint edema was measured by the volume of the paw (mL).

**Results:**

Administration of complete Freund's adjuvant containing *Mycobacterium butyricum* increased paw elevation time and edema in both joints.

**Conclusion:**

These data allow standardization of an animal model of chronic monoarthritis adequate for analysis of the effects of exercise on treatment of rheumatoid arthritis.

## RESUMO

**Objetivo:**

Padronizar um modelo experimental de monoartrite crônica induzida por adjuvante completo de Freund apropriado à análise do efeito da deambulação na nocicepção e no edema articular.

**Métodos:**

Foram avaliados os seguintes fatores para a indução da monoartrite: via e local de administração, número e intervalo das inoculações, espécie de micobactéria e gênero dos animais. Para tanto, ratos Wistar machos e fêmeas (200 a 250g) receberam duas injeções de adjuvante completo de Freund contendo *Mycobacterium tuberculosis* (1,0mg/mL; 50*μ*L) ou *Mycobacterium butiricum* (0,5mg/mL; 50*μ*L) intra-articular nas articulações tibiotársica ou tibiofemural ou, ainda, uma injeção de adjuvante completo de Freund (*Mycobacterium butiricum* ou *tuberculosis*) intradérmica na base da cauda e outra intra-articular (tibiotársica ou tibiofemural). Os animais foram submetidos à avaliação da incapacitação e edema articulares. A incapacitação articular foi avaliada pelo tempo de elevação da pata (em segundos) durante a marcha de 1 minuto. O edema da articulação tibiofemural foi avaliado pela variação do diâmetro articular (cm). O edema da articulação tibiotársica foi medido pelo volume da pata (mL).

**Resultados:**

A administração de adjuvante completo de Freund, contendo *Mycobacterium butiricum*, aumentou o tempo de elevação da pata e o edema, em ambas as articulações.

**Conclusão:**

Esses dados possibilitaram a padronização de um modelo animal de monoartrite crônica, adequado à análise dos efeitos do exercício no tratamento da artrite reumatoide.

## INTRODUCTION

Rheumatoid arthritis (RA) is a chronic and systemic inflammatory process that gradually destroys the synovia and the periarticular structures^(1–3)^. Articular pain and edema are the primary symptoms^(4,5)^, generating limitations in movement, functional restrictions, and decline in musculoskeletal, cardiopulmonary and neuromuscular systems^(6)^. Some studies showed that regular engagement in exercise attenuates the articular inflammatory response, pain, and edema, contributing towards recovery of range of motion, muscle strength, and improvement of cardiovascular condition^(6–8)^; however, its effect has not yet been clearly elucidated.

With the purpose of studying the etiopathogenesis of arthritis and of seeking different treatments, many experimental models representing human arthritis have been developed over the years^(4,9–27)^. Among the experimental models of RA developed^(9–26)^, complete Freund's adjuvant (CFA)-induced arthritis stands out^(9,10–17,19,22,23,25,26)^. In animal models, the manifestation and severity of symptoms may vary according to gender of the animal used, route and site of administration, *Mycobacterium* species, and number and interval of inoculations^(9–17,19,22,23,25,26)^. In this way, performing a study on the effects of exercise on symptoms of adjuvant-induced arthritis require, first of all, standardization of an animal model appropriate for this purpose.

## OBJECTIVE

To standardize an experimental model of CFA-induced chronic monoarthritis in rats, for analysis of the effect of walking on nociception and on articular edema.

## METHODS

### Animals

Thirty Wistar rats were used, 18 of them male, with 60 days of age (200 to 250g), from the Central Animal Laboratory of the Universidade Federal de Santa Catarina (UFSC). The animals were kept under controlled conditions in terms of humidity and temperature (22±2°C), with a 12-hour light/dark cycle, and water and feed *ad libitum*. The experiments were conducted as per ethical guidelines of the International Association for the Study of Pain and approved by the Animal Care and Use Committee of the UFSC (protocol 100/CEUA/PRPe/2007).

### Drugs and reagents

In this study, the following were used: CFA *Mycobacterium tuberculosis* (1 mg/mL, Sigma^®^) or *Mycobabterium butyricum* (0.5mg/mL, Difco^®^); isotonic solution of sodium chloride (0.9%, Aster^®^); halothane gas (250mL, 1:1_v/v_, 2 to 4% diluted in hospital O_2_, Cristália^®^); iodinated alcohol (1%, Rialcool^®^); and aqueous solution of lauryl sulfate (2.5% Vetec^®^).

### Articular disability test

Articular disability was measured by paw elevation time (PET) (in seconds), with the help of a recording system proposed by Tonussi and Ferreira^(21)^, which evaluates nociception. In this analysis, the animals were submitted to forced walking in a steel cylinder (30 x 30cm) at a constant speed (3 rpm) for 60 seconds. Metallic paw covers were adjusted on the hind paws, in which only the right paw was connected to a computer, which recorded the total time that this paw was not in touch with the cylinder surface during the test. One week before the beginning of the experiment, the animals were made accustomed to the walking procedure for a time sufficient for learning to maintain themselves at the top of the moving cylinder. One hour before the test, they were familiarized with wearing the paw covers. The PET of animals with no intra-articular treatment is approximately 10 seconds^(20)^. The increased PET after the intra- articular injection of inflammatory agents indicates the development of articular disability^(20)^.

### Evaluation of articular edema

Evaluation of edema of the tibiofemoral (TF) joint was made with the help of an analogical caliper gauge (0.05mm precision) by recording variation of the articular diameter (AD) in centimeters^(15)^. Evaluation of edema of the tibiotarsal (TT) joint was made with the help of a plastic cuvette filled with lauryl sulfate (2.5%) in water, coupled with precision electronic scales (Acculab, V-121). The animal's paw was submersed until immediately above the TT joint. The dislocation of the liquid column within the cuvette was recorded, in milliliters. Each 1mL of dislocated liquid in the cuvette corresponded to 1 g of weight of the paw^(27)^.

Recordings of PET and edema were made immediately before stimulation with CFA (baseline measurement) and posteriorly until the end of each experiment. Edema measurements were made before the PET analysis.

### Experimental procedures

The study was carried out at the Nociception Neurobiology Laboratory (LANEN) of the Department of Pharmacology of the UFSC. To develop the arthritis model, different procedures were tested, varying route and site of administration (TF, in the suprapatellar ligament region, or TT, lateral joint region); *Mycobacterium* species; the number and interval of inoculations and gender of animals. For this, five experiments were carried out, which evaluated articular disability and edema until the values returned to baseline (end of the experiment).

### Experiment 1

To define the number of CFA administrations necessary to promote chronic arthritis, six male rats received two (intraarticular) injections of CFA containing *Mycobacterium tuberculosis* (1mg/mL; 50*μ*L) into the TF joint with an 8-day interval between injections. The PET and AD were evaluated daily until day 19, and every two days, until day 29 of the experiment.

### Experiment 2

To define the route of administration and the interval between inoculations, six male rats received an intradermal injection of CFA containing *Mycobacterium tuberculosis* (1mg/mL; 50*μ*L) at the base of the tail and in the TF joint (intra-articular, 50*μ*L), after 21 days. PET and AD were evaluated daily until day 28, and every two days until day 42.

To verify differences in behavior of arthritis induction by modifying the gender of the animals, the *Mycobacterium* species used and the type of joint, experiments 3 to 5 were conducted.

### Experiment 3

Six male rats received an injection of CFA containing *Mycobacterium tuberculosis* (1mg/mL; 50*μ*L) at the base of the tail (intradermal) and another in the TT joint (intra-articular, 50*μ*L), after 21 days. PET and edema were evaluated daily until day 28 and every two days, until day 39.

### Experiment 4

Six female rats received an injection of CFA containing *Mycobacterium butyricum* (0.5mg/mL; 50*μ*L) at the base of the tail (intradermal) and another in the TT joint (intra-articular, 50*μ*L), after 21 days. PET and edema were evaluated daily until day 28, and every two days, until day 45.

### Experiment 5

Six female rats received an injection of CFA containing *Mycobacterium butyricum* (0.5mg/mL; 50*μ*L) at the base of the tail (intradermal) and another in the TF joint (intraarticular, 50*μ*L), after 21 days. PET and AD were analyzed daily until day 27, and every two days, until day 44.

### Statistical analysis

The Shapiro-Wilk and Levene tests were applied to verify normality of data and homogeneity of variance, respectively. Student's paired *t* test was used in comparisons between baseline values and those obtained daily for PET and edema. For the analysis, the Statistical Package for the Social Sciences (SPSS) for Windows^®^, version 19.0, was used with a significance level of 5%.

## RESULTS

**Figure 1:**
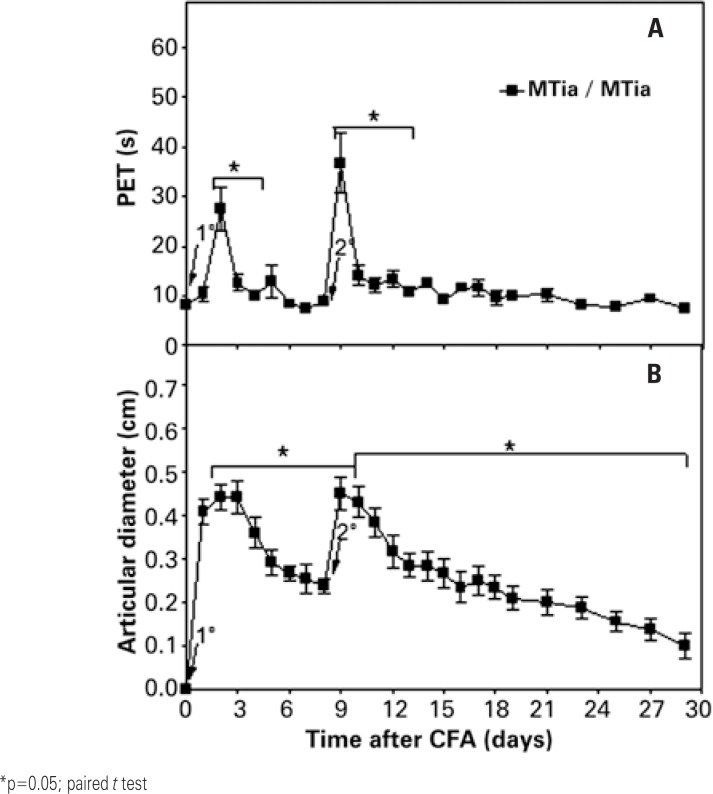
Effect of two intra-articular administrations (intra-articular, days 0 and 8, 1^st^ and 2^nd^ arrows) of complete Freund's adjuvant containing *Mycobacterium tuberculosis* (MT), in the tibiofemoral joint of male rats (MTia/MTia, n=6), on (A) disability and (B) articular diameter

In figure 1, it may be seen that the first administration (intra-articular) of CFA promoted an increase in PET and AD. The peak of the curve occurred on the second day (PET=27.4±4.23 seconds, 234% of the baseline levels; AD=0.44±0.03cm, 44% of the baseline levels). Later there was a drop in disability, with no statistical significance relative to the baseline level as of day 5, returning to 2.02% of the baseline levels on day 6 (PET=8.83±0.33 seconds). AD diminished around day 7 (AD=0.25±0.03cm, 25% above baseline levels), demonstrating a tendency towards maintaining this behavior. Considering that a single administration was not capable to obtain persistent arthritis, a second administration (intra-articular) of CFA was given after 8 days. Twenty-four hours after the second stimulation, there was a new increase in disability (PET=36.68±6.06 seconds, 346% of the baseline levels) and in edema (AD=0.45±0.03cm, 45% of baseline levels). These data evidence the need for two inoculations to promote a considerable effect on parameters PET and AD. However, a decrease in the values of both parameters was noted, losing statistical significance as of day 13 for PET and day 29 for AD.

**Figure 2:**
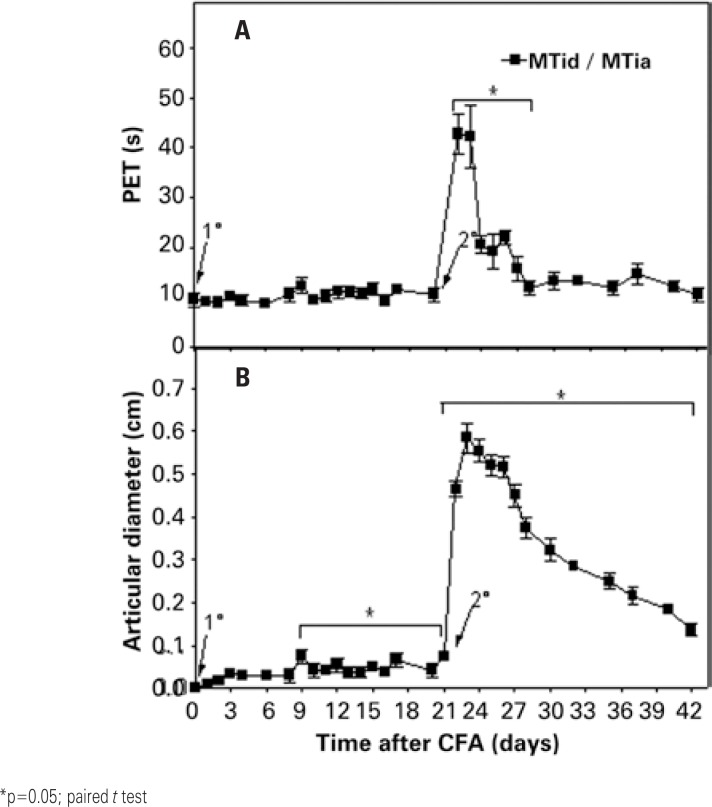
Effect of the administration of complete Freund's adjuvant containing *Mycobacterium tuberculosis* (MT) at the base of the tail (intradermal, day 0, 1^st^ arrow) and in the tibiofemoral joint (intra-articular, day 21, 2^nd^ arrow) of male rats (MTid/MTia, n=6), on (A) disability and (B) articular diameter

Figure 2 shows the results of investigating route of administration and interval between inoculations on the parameters evaluated. The first injection of CFA (intradermal) altered significantly only the values of AD (as of day 9). However, the second stimulation with CFA (intra-articular) promoted an elevation of PET and AD, with a maximal level observed on day 22 for disability (PET=42.70±3.98 seconds, 356% of the baseline levels) and on day 23 for edema (AD=0.58±0.03cm, 58% of the baseline levels). Next, progressive decrease in disability was seen, losing statistical significance as of day 28. On the other hand, despite the gradual decrease observed in AD, the mean values showed significant differences relative to the baseline levels until the end of the experiment. Comparing these results with those of the previous experiment, it was noted that after the second CFA inoculation, PET was slightly more elevated and lasting. As to AD, it was noted that the intradermal injection of CFA caused sensitization of the animals, promoting a significant increase of the effect for the second inoculation. With these data, the interval (21 days) was standardized, and the induction route of arthritis (one intradermal CFA administration followed by another, intra-articular).

**Figure 3:**
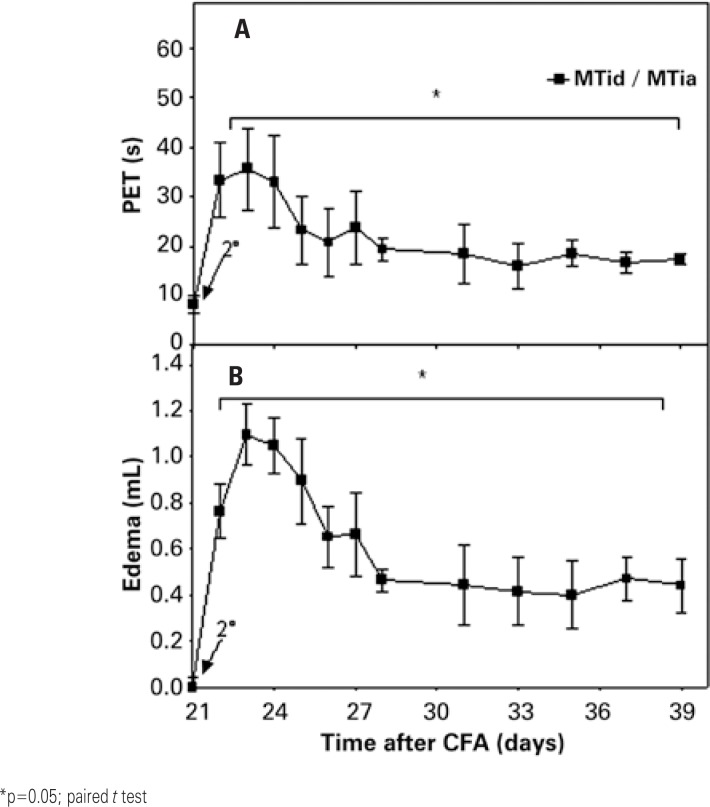
Effect of the administration of complete Freund's adjuvant containing *Mycobacterium tuberculosis* (MT) at the base of the tail (intradermal, day 0), and in the tibiotarsal joint (intra-articular, day 21, 2^nd^ arrow), of male rats (MTid/MTia, n=3), on (A) disability and (B) articular edema

Figure 3 shows data from the investigation of the most adequate joint for chronic arthritis induction. It was noted that the second stimulation with CFA in the TT joint promoted increase in PET and edema as of day 22, with maximal values on day 23 (PET=35.58±8.23 seconds, 209% of the baseline levels; edema=1.09±0.13mL, 109% of the baseline levels). Later, a progressive decline was seen in the values of PET and edema, losing statistical significance as of day 37. Nevertheless, edema with necrotic aspect was seen in three animals, which were euthanized before the conclusion of the experiment. Comparing these results with those obtained in experiment 2, it was clear that for this *Mycobacterium* (*Mycobacterium tuberculosis*) the TT joint was less appropriate, producing severe arthritis relative to the TF joint.

**Figure 4:**
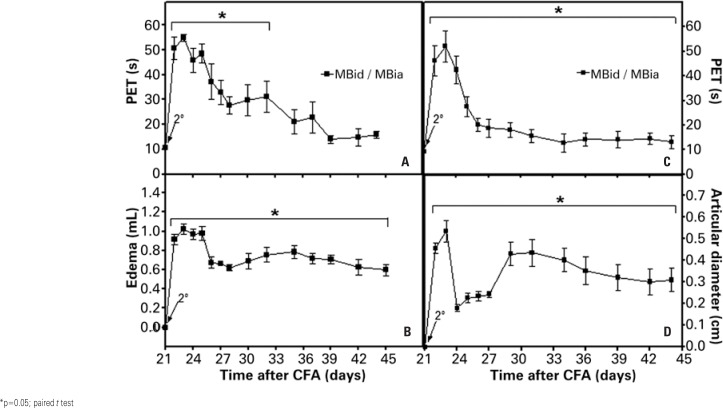
Effect of the administration of complete Freund's adjuvant containing *Mycobacterium butyricum* (MB) with the second stimulation (intra-articular, day 21, 2^nd^ arrow) into the tibiotarsal joint (left side: A and B) or in the tibiofemoral joint (right side: C and D) of female rats (MBid/MBia, n=6), on disability (above: A and C) and edema (below: articular edema in B; articular diameter in D)

The evaluation of the effect of the *Mycobacterium* species and of gender of the animals on the parameters of PET and edema are illustrated in figure 4. In this experiment, it was only after the second stimulation with CFA that a significant increase in PET and edema was observed. These values reached maximal levels on day 23 (PET=54.8±1.40 seconds, 319% of the baseline levels; edema=1.02±0.05mL, 188% of the baseline levels), reduced after day 25. A significant loss of statistical significance was observed relative to the baseline values as of day 35 of the experiment for PET. As for edema, despite displaying a decrease in values, these remained significantly higher than the baseline values until the end of the experiment (Figures 4A and 4B). With the use of females and of *Mycobacterium butyricum* it was possible to observe a lasting induction of monoarthritis for the TT joint, with less severe characteristics than those seen in experiment 3.

Figures 4C and 4D show the result of the investigation of monoarthritis by *Mycobacterium butyricum* in the TF joint in females. It was noted that the second stimulation with CFA promoted increased PET and AD. These values reached maximal levels on day 23 of the experiment (PET=51.3±6.22 seconds, 467% of the baseline levels; AD=0.53±0.04mL, 53% of the baseline levels), with a reduction initiated on day 24. However, the values in both parameters showed significant differences relative to the respective baseline values until the end of the experiment (day 44). Such results indicate that the second injection in the TF joint provoked lasting arthritis, since although the results of the disability test indicated that nociception was reduced throughout the study, edema remained stable until the end of the experiment.

## DISCUSSION

According to Wauben et al.^(22)^, the ideal dose for induction of severe arthritis is 0.1mL of *Mycobacterium* at 10.0mg/mL and, for less severe cases, 1.0 to 5.0mg/mL. Indications of adjuvant volume, of the administration route, and of the joint used in rats varied among the studies: Nagakura et al.^(17)^and Cook and Moore^(12)^ injected 100*μ*L of CFA (5.0mg/mL, *Mycobacterium butyricum*) intraplantar; Zhang et al.^(25)^ utilized 100*μ*L of CFA (0.5mg/mL, *Mycobacterium tuberculosis*) at the base of the tail, intradermal; Zheng et al.^(26)^ used 100*μ*L of adjuvant, (10mg/mL, *Mycobacterium tuberculosis*), also at the base of the tail, intradermal; Yu et al.^(4)^ utilized 125*μ*L of CFA (250*μ*g, *Mycobacterium butyricum*), in the knee; Sharif Naeini et al.^(18)^ injected 25*μ*L of CFA (135*μ*g/mL, *Mycobacterium butyricum*) in the ankle joint. All achieved adjuvant induction of arthritis meeting their purposes.

In this study, the concentration of CFA, according to the type of *Mycobacterium* and the volume injected, took into consideration the particularities of the articular disability test (forced walking). Induction of severe arthritis would hinder the performance of the test, but arthritis needed to endure to allow evaluation of treatment in future studies.

Initially, the need for two intra-articular inoculations of CFA to promote a considerable effect on the parameters PET and AD was determined. The literature shows that the administration of 100 and 150*μ*L of CFA (1mg/mL, *Mycobacterium tuberculosis*) in the knee joint causes hyperalgia and edema for 14 and 90 days, respectively^(24)^. Yu et al.^(4)^ administered 125*μ*L of CFA (250*μ*g, *Mycobacterium butyricum*) into the knee joint, and observed hyperalgesia and edema for 20 to 30 days. Additionally, Donaldson et al., demonstrated that the intra-articular effects of CFA are dose-dependent^(14)^. Despite the difference in concentration and in the volume of administration of CFA relative to the present study, these authors did not analyze spontaneous pain upon movement.

The use of one injection of CFA (*Mycobacterium tuberculosis*) at the base of the tail (intradermal) was based on the findings of Wauben et al.^(22)^, which suggest this as the preferential route of immunization. However, this did not promote significant signs of pain and edema. It was only after the second CFA injection, into the TF joint, that a more lasting PET and an elevated AD were noted. On the other hand, in the TT joint, there was edema with necrotic aspect on the paw. Therefore, the injection of CFA *Mycobacterium tuberculosis* into the TT joint was less adequate relative to the TF joint since it produced a less severe arthritis. Cai et al.^(11)^and Zheng et al.^(26)^ induced arthritis by a single 100*μ*L intradermal injection of CFA (5.0mg/mL*, Mycobacterium tuberculosis*) at the base of Lewis rat tails. Nevertheless, these authors did not describe in detail the manipulation techniques and doses of *Mycobacterium tuberculosis* that influenced induction and severity of induction of arthritis^(11,26)^.

The results obtained to verify the effect of the *Mycobacterium* species and of gender of the animals on the parameters studied again demonstrated a significant increase in PET and edema only after the second stimulation, in both joints. Banik et al.^(28)^ demonstrated that the inflammatory and arthritic signs, after injection of *Mycobacterium butyricum* at the base of the tail, appeared earlier, and were more severe and consistent in Lewis rats. However, Costa et al.^(13)^ carried out an induction of arthritis with an injection of 50*μ*L of CFA (5mg/mL, *Mycobacterium butyricum*) at the base of the tail of Wistar rats, observing edema for 25 days in the TT joint. Similar results of hyperalgesia and edema were found by Sharif Naeini et al.^(18)^, after an injection of 25*μ*L of CFA, containing 135*μ*g/mL of *Mycobacterium butyricum*, into the TT joint^(18)^.

With the use of females and of *Mycobacterium butyricum,* it was possible to observe the induction of a lasting monoarthritis for the TT and TF joints, with less severe characteristics than those observed with *Mycobacterium tuberculosis* and with the use of males. The influence of gender was reported in some studies for some breeds of rats, but there is no significant restriction of gender on susceptibility regarding arthritis^(11,22)^.

As to the number of inoculations, Buttler et al.^(10)^ administered six CFA injections (300mg/0.05mL, *Mycobacterium butyricum)* at the TT joint, suggesting this model for studies of chronic arthritis and reinforcing the need to perform more than one inoculation with CFA for the development of long-term studies. However, two doses of CFA (*Mycobacterium butyricum*) were sufficient to increase PET and edema in a persistent manner, in both joints studied. These data allow the standardization of an animal model of arthritis induced by CFA, adequate for analysis of the effect of walking on nociception and on articular edema, enabling the development of future studies to evaluate exercise in treating RA.

## CONCLUSION

Based on the results obtained, it was noted that the model of CFA-induced arthritis that is adequate for analysis of the effect of walking on nociception and on articular edema displays the following characteristics: two inoculations of CFA containing *Mycobacterium butyricum,* where the first injection is intradermal and performed at the base of the tail 21 days before the second, which is intra-articular, into the TF or TT joint, both in male and female Wistar rats. Considering that the articular disability tests and the measurement of edema are important in the evaluation of effectiveness of exercise as a treatment of arthritis, the standardization of the model adequate for these analyses is relevant to our purposes. We conclude that this animal model of arthritis induction is adequate for the study on walking, allowing the development of future studies for evaluation of exercise in the treatment of RA.
